# Supplementary material for: k-mer manifold approximation and projection for visualizing DNA sequences
Source: Genome Res. 2025 May;35(5):1234–46. doi: 10.1101/gr.279458.124 (PMC12047656; doi:10.1101/gr.279458.124)
Supplement: Supplement 8 [file Supplemental_Data_1.zip › Supplemental_Data_1/Tcfap2a_TGATCC20NCG_P_3/Tcfap2a_TGATCC20NCG_P_3_UMAP.pdf]

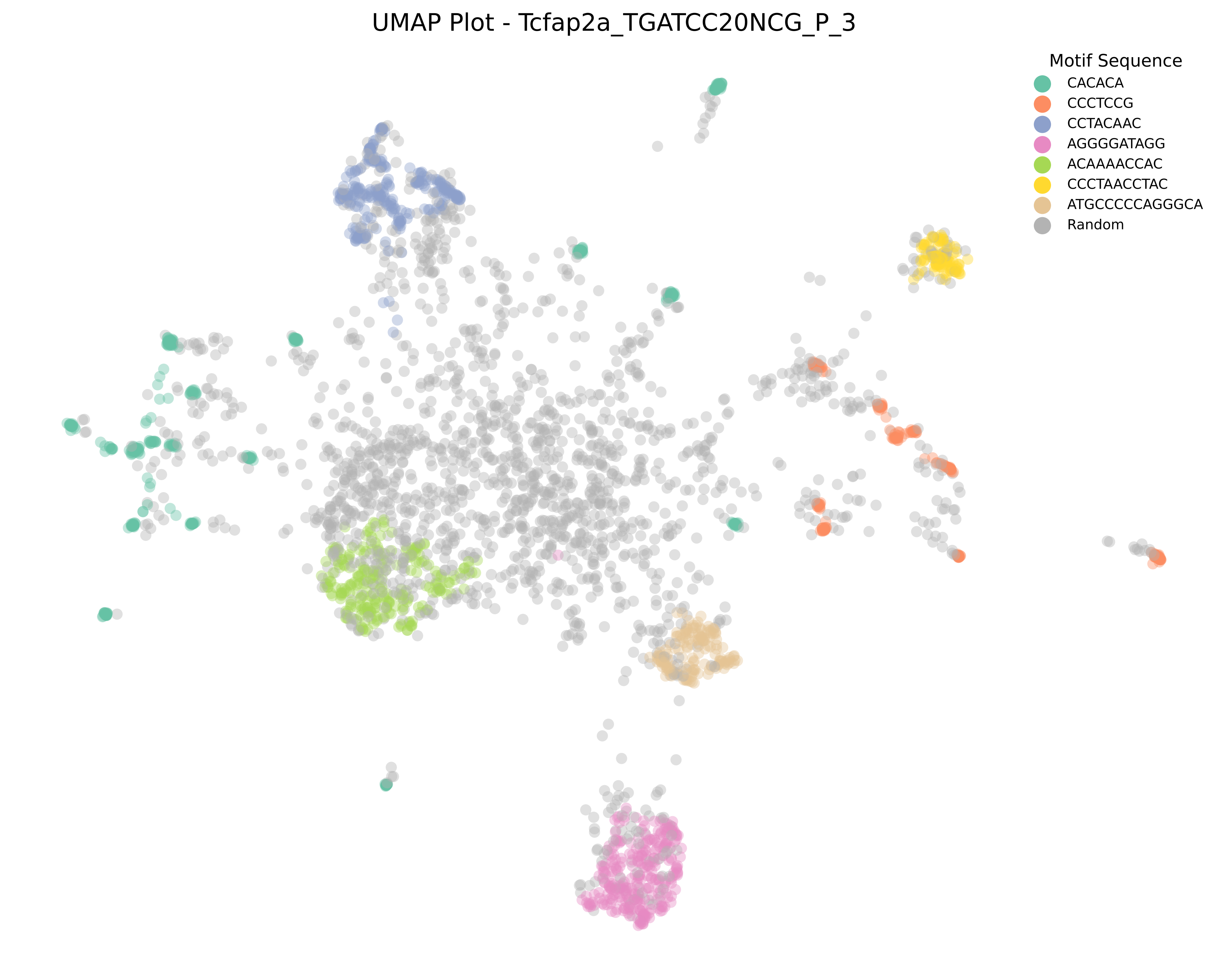
UMAP Plot - Tcfap2a_TGATCC20NCG_P_3
Motif Sequence
CACACA
CCCTCCG
CCTACAAC
AGGGGATAGG
ACAAAACCAC
CCCTAACCTAC
ATGCCCCCAGGGCA
Random
